# Supplementary material for: Dynamic Succession of Microbiota during Ensiling of Whole Plant Corn Following Inoculation with Lactobacillus buchneri and Lactobacillus hilgardii Alone or in Combination
Source: Microorganisms. 2019 Nov 21;7(12):595. doi: 10.3390/microorganisms7120595 (PMC6955939; doi:10.3390/microorganisms7120595)
Supplement: Supplementary file 1 [file microorganisms-07-00595-s001.pdf]

microorganisms

Supplementary Materials

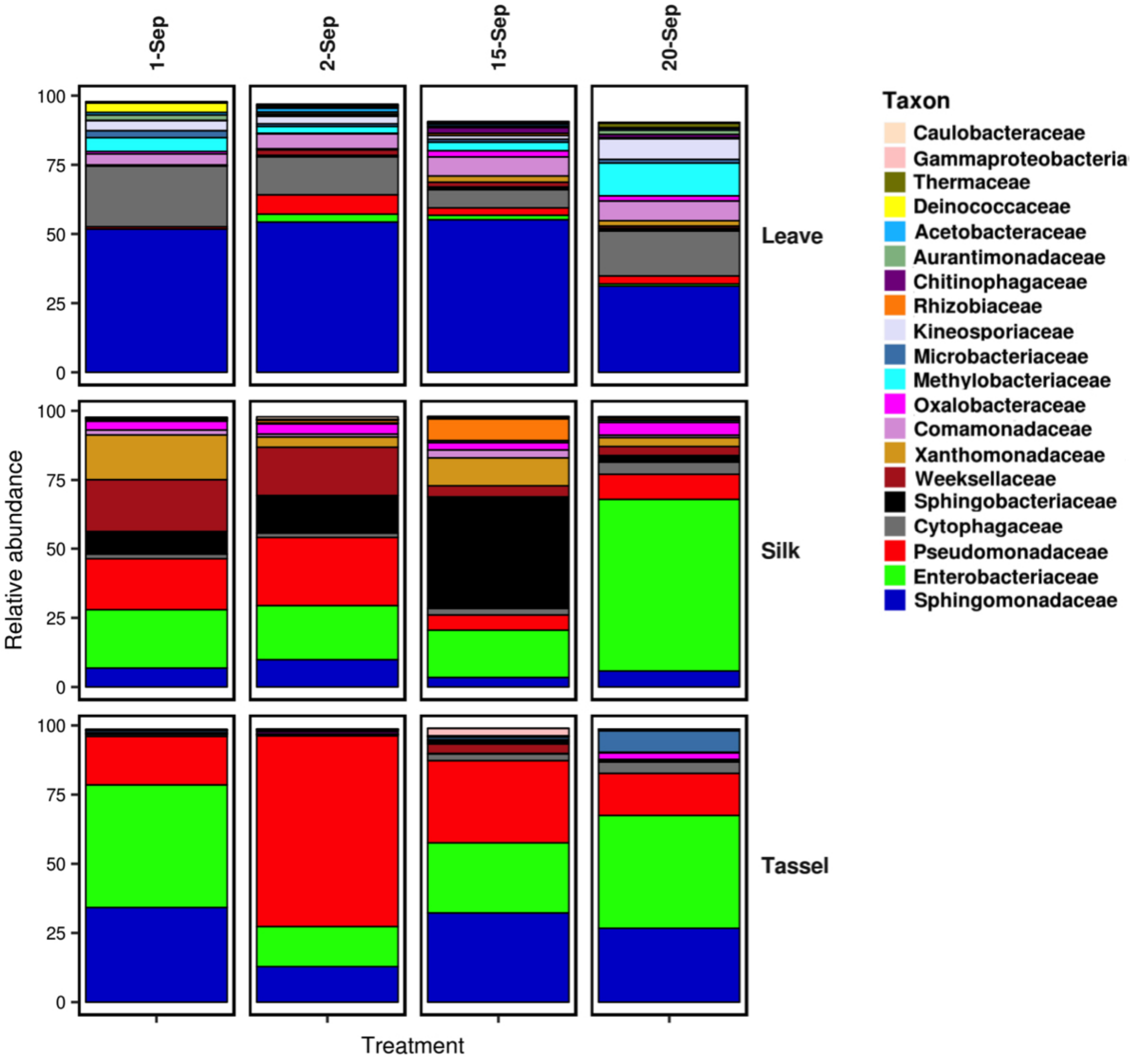

Figure S1. 16S Amplicon metasequencing diversity profile of corn at four sampling periods in the month preceding harvest. Colour coding of relative abundance data is at the family level. The DNA of individual samples of leaves, silk plus a few terminal kernels, and tassels extracted and sequenced individually. Main weather details: it rained on the day preceding 1-Sept. sampling; all the other days preceding sampling were sunny.

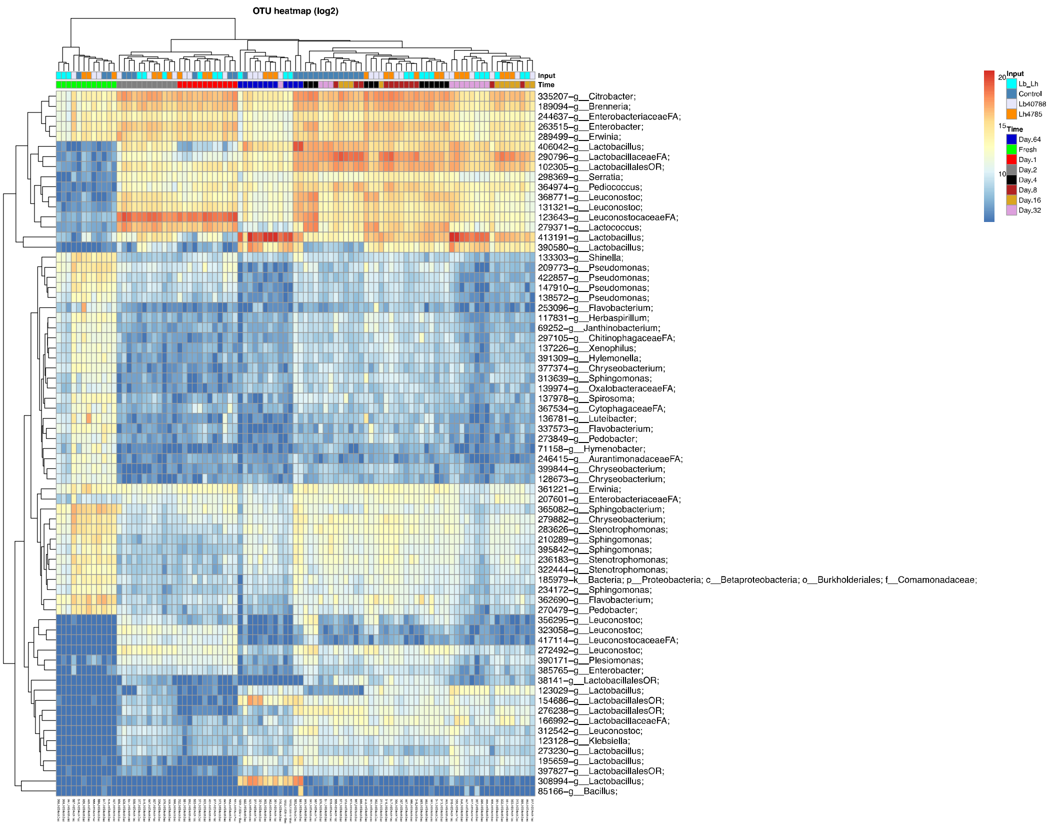

Figure S2. Heatmap and cladogram of the samples for the 16S amplicon metasequencing (V4 region) data from corn silages inoculated with four inoculation treatments and eight fermentation periods, ranging from fresh to 64 days of ensiling. Color scale represents the $\log_2$ transformation of the CPM (count per million reads).

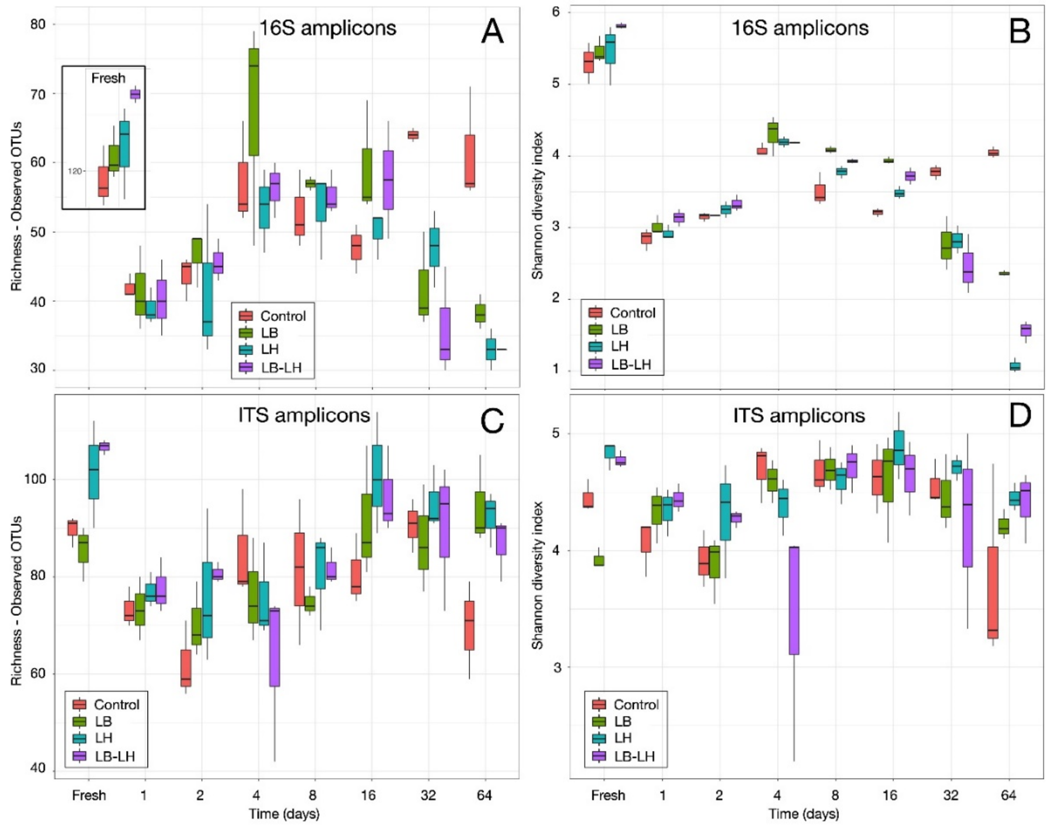

Figure S3. Changes in alpha diversity parameters, Shannon Index and Observed Species, in relation to time of fermentation for the 16S and the ITS amplicons for the four treatments.

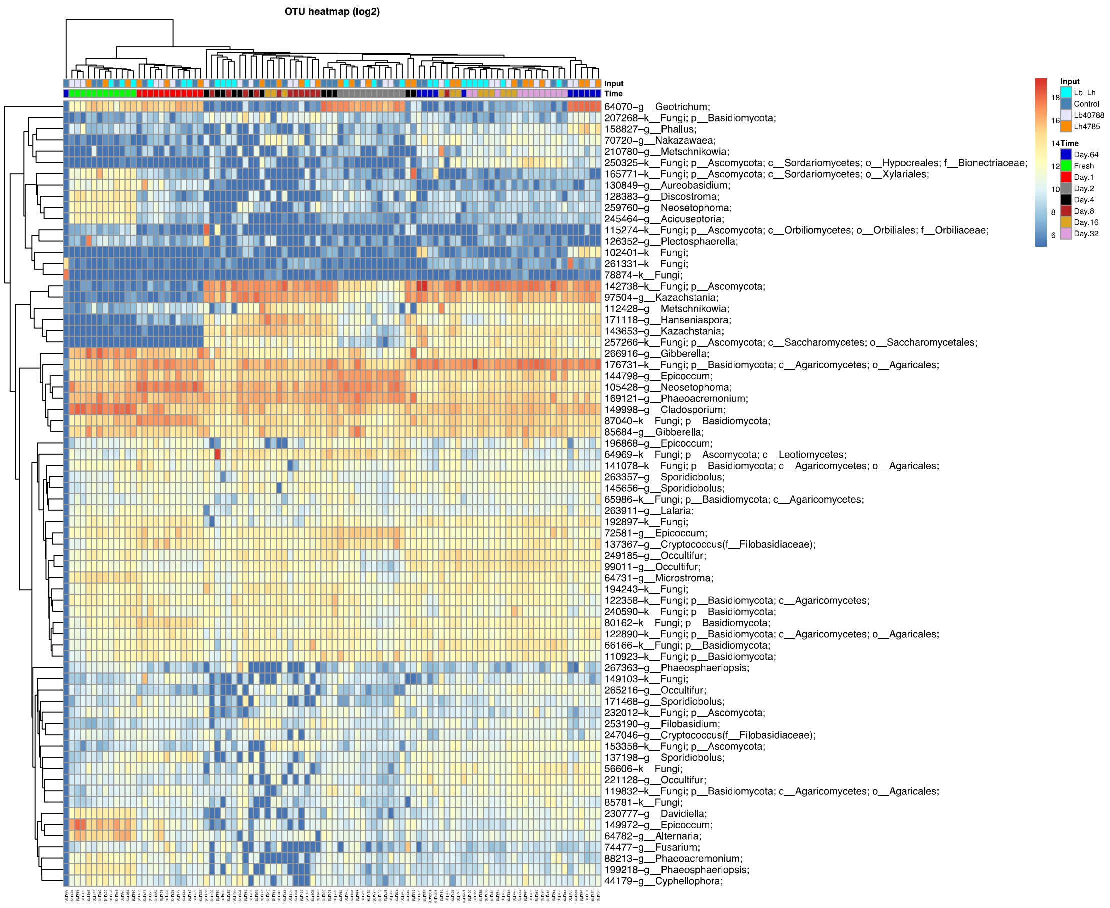

Figure S4. Heatmap and cladogram of the samples for the ITS amplicon metasequencing data from corn silages inoculated with four inoculation treatments and eight fermentation periods, ranging from fresh to 64 days of ensiling. Color scale represents the $\log_2$ transformation of the CPM (count per million reads).

Table S1. Table identifying the three main OTUs that increased and the three that decreased between inoculation versus Control (LB-LH over Control, LB over Control, and LH over Control) of whole plant corn silage. Taxonomic identification was performed using the contrast results (EdgeR package) following a BLASTn search.

Table S1. Taxonomic identification (BLASTn against NCBI GenBank database) of the three top OTU showing highest increase and highest decrease following results by a contrast analysis of the bacteria population after inoculation with LB, LH, or LB-LH against the control treatment, by individual opening period.

Day(s)	Treatments					
	LB-LH over control		LB over control		LH over control	
	Higher in control	Higher with inoculation	Higher in the control	Higher with inoculation	Higher in control	Higher with inoculation
1	None	LH LD LF (4.20) [3]	*Lactobacillus brevis* (-2.93)<br>*Lactobacillus plantarum* (-2.89)<br>*Pediococcus pentosaceus* (-2.81)	Uncultured bacterium (4.67)	None	LH LD LF (4.00)
2	None	*Lactobacillus buchneri* (5.64)<br>*Lactobacillus buchneri* (4.04)<br>LH LD LF (3.47)	*Lactobacillus pentosiphilus* (-2.93)<br>*Lactobacillus sharpeae* (-2.83)<br>*Lactobacillus parabrevis* (-2.63)	*Lactobacillus buchneri* (3.92)<br>LH LD LF (1.62)	*Leuconostoc fallax* (-2.63)	LH LD LF (3.22)<br>*Lactobacillus buchneri* (3.09)
4	*Leuconostoc pseudomesenteroides* (-4.78) [1]<br>*Pediococcus acidilactici* (-4.61) [2]<br>*Lactobacillus* (uncultured) (-4.23)	*Lactobacillus buchneri* (6.82)<br>*Lactobacillus buchneri* (4.08)<br>*Flavobacterium* sp. (3.39)	*Pediococcus acidilactici* (-3.36)<br>*Weisella confusa* (-3.09)<br>*Lactobacillus plantarum* (-3.09)	*Lactobacillus buchneri* (6.58)<br>*Lactobacillus buchneri* (3.95)<br>*Flavobacterium phychrophilum* (3.93)	Uncultured *Lactobacillus* (-4.02)<br>*Pediococcus acidilactici* (-3.88)<br>*Lactobacillus parabrevis* (-3.80)	*Lactobacillus buchneri* (4.85)<br>*Luteibacter rhizovicinus* (4.50)<br>*Lactobacillus buchneri* (3.73)
8	*Lactobacillus pentosiphilus* (-2.38)<br>*Burkholderia gladioli* (-2.30)<br>*Lactobacillus brevis* (-1.69)	*Lactobacillus buchneri* (5.08)<br>*Lactobacillus buchneri* (4.51)<br>Uncultured bacterium (2.89)	*Lactobacillus brevis* (-3.39)<br>*Lactobacillus pentosiphilus* (-3.03)<br>*Lactobacillus brevis* (-2.52)	*Lactobacillus buchneri* (4.96)<br>*Lactobacillus buchneri* (4.42)<br>*Hafnia* sp. (1.94)	None	*Lactobacillus buchneri* (4.92)<br>*Lactobacillus buchneri* (4.57)<br>LH LD LF (2.80)
16	*Lactobacillus brevis* (-2.14)	*Lactobacillus buchneri* (5.64)<br>*Lactobacillus buchneri* (4.04)<br>LH LD LF (3.47)	*Spiromonas radiotolerans* (-3.95)<br>*Lactobacillus pentosiphilus* (-2.30)<br>*Pediococcus pentosaceus* (-2.15)	*Lactobacillus buchneri* (7.49)<br>*Lactobacillus buchneri* (6.58)<br>LH LD LF (3.52)	None	*Lactobacilus buchneri* (6.73)<br>*Lactobacillus buchneri* (6.07)<br>LH LD LF (3.78)
32	*Siphonobacter* sp. (-4.23)<br>*Lactobacillus brevis* (-4.16)<br>*Xanthobacter autotrophicus* (-3.99)	*Lactobacillus buchneri* (7.52)<br>*Lactobacillus buchneri* (5.84)<br>LH LD LF (5.41)	*Lactobacillus pentosiphilus* (-3.37)<br>*Spiromonas radiotolerans* (-2.65)<br>*Lactobacillus plantarum* (-1.94)	*Lactobacillus buchneri* (8.45)<br>*Lactobacillus buchneri* (6.80)<br>LH LD LF (6.25)	*Hymenobacter* sp. (-3.39)<br>*Lactobacillus brevis* (-2.97)<br>*Spingomonas roseiflava* (-2.54)	*Lactobacillus buchneri* (6.67)<br>*Lactobacillus buchneri* (6.43)<br>LH LD LF (6.32)
64	*Bacillus pumilis* (-8.69)<br>*Lactobacillus pentosiphilus* (-5.56)<br>*Leuconostoc lactis* (-5.32)	LH LD LF (2.73)<br>*Lactobacillus helveticus* (2.12)<br>*Lactobacillus buchneri* (1.97)	*Bacillus pumilis* (-8.69)<br>*Lactobacillus pentosiphilus* (-5.51)<br>*Leuconostoc lactis* (-5.31)	*Lactobacillus ingluviei* (5.73)<br>*Pediococcus damnosus* (3.22)<br>*Lactobacillus harbinensis* (3.16)	*Bacillus pumilis* (-8.69)<br>*Lactobacillus pentosiphilus* (-5.85)<br>*Leuconostoc lactis* (-4.94)	LH LD LF (3.69)<br>*Lactobacillus helveticus* (3.56)

1. Values in parenthesis represent the logFC (fold change) generated by the contrast analysis performed using the EdgeR package. Negative numbers represent OTU more present in the control samples.
2. Taxonomic identification represents the main identify level of the top 10 to 20 results of the BLASTn search.
3. LH LD LF OTU were identical between *Lactobacillus hilgardii*, *Lactobacillus diolivorans*, and *Lactobacillus farraginis* sequences.

Table S2. Table identifying the four main OTUs that increased and the four that decreased between fermentation periods in the Control, LB, LH, and LB-LH treatments of whole plant corn silage. Taxonomic identification was performed using the contrast results (EdgeR package) following a BLASTn search. In addition to the RDP classifier (methods), OTU taxonomy was further characterized by BLASTing them against NCBI GenBank general nucleotide collection database (nr/nt). Considering the small size of individual OTU, taxonomic identification was good with identical hits showing 100% identity with most of the top 20 hits.

Table S2. Taxonomic identification (BLASTn against NCBI GenBank database) of the four top OTU showing highest increase and highest decrease following results by a contrast analysis of the bacteria population for the four treatments (Control, LB, LH, and combo LB-LH) compared between one opening period and the previous one.

DAY	LB-LH		LB	
	Decrease	Increase	Decrease	Increase
1 over fresh	*Acinetobacter baylyi* (-4.37) [1]<br>*Chryseobacterium* sp. (-4.19)<br>*Herbaspirillum chlorophenolicum* (-3.90)<br>*Luteibacter rhizovicinus* (-3.75)	*Weisella confusa* [2] (10.83)<br>*Lactococcus lactis* (9.40)<br>*Pediococcus pentosaceus* (8.23)<br>*Leuconostoc mesenteroides* (7.88)	*Hymenobacter* sp. (-7.09)<br>*Spirosoma radiotolerans* (-6.50)<br>*Spingomonas* sp. (-6.50)<br>*Brevundimonas* sp. (-6.40)	*Weisella confusa* (10.91)<br>*Lactococcus lactis* (8.72)<br>*Leuconostoc mesenteroides* (7.00)<br>*Pediococcus pentosaceus* (6.63)
2 over 1	*Sphingobacterium* sp. (-1.90)<br>*Chryseobacterium* (-1.74)<br>*Pediococcus pentosaceus* (-1.68)	*Lactobacillus brevis* (4.74)<br>*Lactobacillus buchneri* (3.64)<br>*Lactobacillus pentosiphilus* (3.34)<br>LH LD LF [3] (3.04)	*Spingobacteria* (unculcultured) (-5.15)	*Lactobacillus brevis* (5.48)<br>LH LD LF (5.01)<br>*Lactobacillus buchneri* (4.55)<br>*Leuconostoc citreum* (2.98)
4 over 2	*Leuconostoc pseudomesenteroides* (-4.09)<br>*Weissella confusa* (-3.80)<br>Uncultured *Lactobacillus* (-3.53)<br>*Weisella cibaria* (-3.49)	Uncultured (*Spingobacterium*) (5.31)<br>*Flavobacterium johnsoniae* (5.02)<br>*Lactobacillus plantarum* (4.06)<br>*Lactobacillus crustorum*	*Weisella confusa* (-3.50)<br>*Weisella confusa* (-3.15)<br>*Weisella* sp. (-3.05)<br>*Weisella cibaria* (-2.97)	*Xanthomonas* sp. (5.30)<br>*Pseudoxanthomonas mexicana* (5.29)<br>*Flavobacterium johnsoniae* (5.27)<br>*Sodalis* sp. (4.90)
8 over 4	*Xanthomonas* sp. (-3.50)<br>*Leuconostoc citreum* (-3.24)<br>*Pseudomonas* sp. (-2.19)<br>*Lactococcus lactis* (-2.04)	None	*Leuconostoc pseudomesenteroides* (-3.16)<br>*Leuconostoc citreum* (-2.61)	None
16 over 8	*Leuconostoc citreum* (-2.70)<br>*Lactococcus lactis* (-2.65)<br>*Pediococcus pentosaceus* (-2.12)<br>*Leuconostoc pseudomesenteroides* (-2.03)	*Lactobacillus buchneri* (1.74)	None	None
32 over 16	*Hafnia alvei* (-2.98)<br>*Kluyvera georgiana* (-2.55)<br>*Variovorax* sp. (-2.26)<br>*Enterobacter* sp. (-2.25)	LH LD LF (2.20)<br>Uncultured (1.79)<br>*Lactobacillus buchneri* (1.56)	None	*Lactobacillus acetotolerans* (7.02)<br>LH LD LF (3.15)<br>*Lactobacillus pentosiphilus* (2.45)
64 over 32	*Lactobacillus plantarum* (-3.08)<br>*Lactobacillus plantarum* (-2.61)<br>*Lactobacillus buchneri* (-2.43)<br>*Lactobacillus pentosiphilus* (-2.26)	*Lactobacillus acetotolerans* (9.52)<br>*Lactobacillus helveticus* (4.73)<br>*Lactobacillus brevis* (3.51)<br>*Lactobacillus pentosiphilus* (3.22)	*Leuconostoc citreum* (-3.59)<br>*Weisella confusa* (-3.02)<br>*Leuconostoc* sp. (-3.02)<br>*Leuconostoc citreum* (-2.95)	*Lactobacillus ingluviei* (5.74)<br>*Lactobacillus acetotolerans* (4.90)<br>*Lactobacillus helveticus* (4.85)<br>*Lactobacillus brevis* (4.61)

DAY	LH		CONTROL	
	Decrease	Increase	Decrease	Increase
1 over fresh	*Luteibacter rhizovicinus* (-9.29)<br>*Flavobacterium* sp. (-8.97)<br>*Dyadobacter* sp. (-8.17)<br>*Spingobacterium* sp. (-7.54)	*Weisella confusa* (10.37)<br>*Lactococcus lactis* (9.23)<br>*Pediococcus pentosaceus* (8.32)<br>*Leuconostoc mesenteroides* (7.30)	*Flavobacterium akiainvivens* (-6.88)<br>*Chryseobacterium* sp. (-5.59)<br>Uncultured *Flavobacterium* (-5.24)<br>*Flavobacterium psychrophilum* (-5.20)	*Weisella confusa* (11.49)<br>*Lactococcus lactis* (9.97)<br>*Leuconostoc mesenteroides* (8.73)<br>*Pediococcus pentosaceus* (8.50)
2 over 1	None	*Lactobacillus brevis* (5.62)<br>*Lactobacillus pentosiphilus* (3.56)<br>LH LD LF (2.98)<br>*Lactobacillus buchneri* (2.53)	*Siphonobacter* sp. (-3.40)<br>*Agrobacterium tumefaciens* (-2.81)	*Lactobacillus brevis* (5.14)<br>*Lactobacillus pentosiphilus* (4.13)<br>LH LD LF (3.73)<br>*Lactobacillus crustorum* (3.54)
4 over 2	*Lactobacillus plantarum* (-3.54)<br>*Leuconostoc* sp. (-3.18)<br>*Weisella confusa* (-3.11)<br>*Leuconostoc pseudomesenteroides* (-3.08)	*Luteibacter rhizovicinus* (6.02)<br>*Lactobacillus plantarum* (4.65)<br>*Spingomonas faeni* (4.64)<br>*Duganella* sp. (3.92)	None	*Lactobacillus plantarum* (4.06)<br>*Lactobacillus plantarum* (3.94)<br>*Lactobacillus pentosiphilus* (3.40)<br>Uncultured *Lactobacillus* (3.35)
8 over 4	*Xanthomonas* sp. (-3.26)<br>*Leuconostoc citreum* (-2.98)<br>*Leuconostoc pseudomesenteroides* (-1.91)	*Lactobacillus plantarum* (1.67)	*Leuconostoc pseudomesenteroides* (-5.12)<br>*Leuconostoc citreum* (-5.05)<br>*Lactobacillus plantarum* (-3.81)<br>*Weisella confusa* (-3.72)	*Lactobacillus plantarum* (1.96)<br>*Lactobacillus sharpeae* (1.91)
16 over 8	*Lysobacter firmicutimachus* (-3.54)<br>*Lactococcus lactis* (-3.41)<br>*Lactobacillus sharpeae* (-3.18)<br>*Herbaspirillus chlorophenolicum* (-3.11)	None	None	None
32 over 16	None	Uncultured bacterium (3.14)<br>LH LD LF (2.96)<br>*Leuconostoc citreum* (2.45)<br>*Leuconostoc citreum* (2.38)	*Lactobacillus sharpeae* (-2.66)	None
64 over 32	*Lactobacillus pentosiphilus* (-3.98)<br>*Lactobacillus plantarum* (-3.89)<br>Uncultured Lactobacillus (-3.58)<br>*Lactobacillus buchneri* (-3.45)	*Lactobacillus acetotolerans* (8.98)<br>*Lactobacillus helveticus* (4.25)	None	*Lactobacillus acetotolerans* (10.51)<br>*Bacillus aerius* (8.27)<br>*Lactobacillus buchneri* (5.98)<br>*Lactobacillus helveticus* (5.20)

1. Values in parenthesis represent the logFC (Fold Change) generated by the contrast analysis performed using the EdgeR package of R. Negative numbers represent OTU more present in the previous opening.
2. Taxonomic identification represents the main identity level observed in the top 10 to 20 results of the BLASTn search.
3. LH LD LF OTU provided similar results between *Lactobacillus hilgardii*, *Lactobacillus diolivorans*, and *Lactobacillus farraginis* sequences in the database.

© 2019 by the authors. Submitted for possible open access publication under the terms and conditions of the Creative Commons Attribution (CC BY) license (http://creativecommons.org/licenses/by/4.0/).
